# Determinants of Health Management Information Systems performance: lessons from a district level assessment

**DOI:** 10.1186/1753-6561-6-S5-O17

**Published:** 2012-09-28

**Authors:** Amit Mishra, Itisha Vasisht, Alia Kauser, Sundararaman Thiagarajan, Dilip Singh Mairembam

**Affiliations:** 1 National Health System Resource Centre, Delhi, India

## Introduction

Developing Health Management Information Systems (HMIS) is challenging in a developing world context where health professionals and managers face complex socio-political and health system challenges. In India, a nation-wide HMIS was put in place under the National Rural Health Mission in 2008. Currently, this system provides monthly reports on over 300 data elements from all the 642 districts in the country. However, the system’s performance in terms of use of information lags behind expectations. Issues around data quality and institutional capacity have also been a concern. This study has been conducted with the objective of understanding the determinants of data quality and use of information at district level.

## Methods

Data for this study were collected from 35 districts of 18 states in May-December 2011. Multistage random sampling was used to identify two districts in each state and ten facilities covering all levels of public health facilities in each district. Data status and flow at the block and district level were also assessed. Primary data of the study was collected by administering a semi structured questionnaire to program managers and data managers at district and block level and auxiliary nurse mid-wives at the facility level. In addition, data collection and reporting processes were closely observed by the research team. Secondary data were collected from records at health facilities in blocks and districts.

## Results

All 35 districts had reported regularly for all months in the study period and had adequate human resources and computers with internet connectivity. Training had been received by the auxiliary nurse midwives (88% of districts), data entry operators (100% of districts) and by all users of the information system (29% of districts). In only 6% of districts, program managers were trained on the use of information. Other than data entry operators, no other category of staff received any refresher training.

Error management protocols and data validation protocols have been established in 20% of districts. No district had developed protocols for non-reporting, incomplete reporting, bulk reporting and post data entry error management. 18% of districts established institutional structures for management of the information system at district level in the form of HMIS teams and 23% of districts made institutional arrangements for data collection from private sector.

In 77% of districts, facility registers were not compatible with the system’s data requirements. In 85% of districts, district hospitals used handmade unformatted registers where data extraction was difficult.

In 52% of districts, district hospital managers were unaware of the reporting requirements. Pre- existing reports on reproductive and child health were still used parallel to HMIS in 77% of districts with conflicting data collection processes. 43% of districts had started giving feedback to the block on monthly basis. However indicators based feedback has started in only 11% of districts. We observed little discrepancy between reported and recorded data.

## Discussion

Poor quality of data is often perceived as largely being a matter of better enforcement of the rules, or of computer related skills or even disciplinary action against false reporting. Our study shows, that these are not serious limitations. The problems of quality data are due to operational design constraints in work organization at each stage of information flow: - in the collection, processing, reporting and analysis of data. Managerial capacity and the capacity to use information lags behind in places where there are deficits in capacity related to human resource, computers and data entry skills. These deficits get compounded by the design features in the software, which are not friendly to local use of information. Institutional capacity gaps need to be addressed through incremental innovation and improvements to maximize the use of information through the HMIS.

## Competing interests

Authors declare that they have no conflict of interest.

## Funding statement

This study was funded by the National Health Systems Resource Centre, New Delhi.

